# Mood dysregulation

**DOI:** 10.1007/s00787-012-0355-9

**Published:** 2012-12-11

**Authors:** Nina Mikita, Argyris Stringaris

**Affiliations:** Institute of Psychiatry, King’s College London, Box P085, De Crespigny Park, Denmark Hill, London, SE5 8AF UK

**Keywords:** Irritability, Hypomania, Bipolar disorder, Nosology, Severe mood dysregulation

## Abstract

The publication of the DSM-5 is nearing, yet a debate continues about the boundaries of bipolar disorder (BP) in children and adolescents. This article focuses on two key components of this debate that are often treated under the collective term mood dysregulation: the first is chronic irritability (and the proposed DSM-5 category of disruptive mood dysregulation disorder) and the other concerns short episodes of mania-like symptoms. We update our previous review [Stringaris in Eur Child Adolesc Psychiatry 20(2):61–66, 2011] and also present relevant neurobiological evidence. Most findings so far suggests that chronic, severe irritability is not a developmental presentation of mania. The diagnostic status of brief duration hypomania is less clear, with some evidence in support of its clinical relevance to BP. We end with recommendations for future research to inform classification and treatment.

## Association between irritability and bipolar disorder (BP)

### Overview on irritability and the bipolar debate

The motivation for research in irritability originates in the so-called bipolar debate. Over the last 15 years, the rates of BP diagnoses in children and adolescents in the USA have increased dramatically, both in inpatient [2] and outpatient services [3]. The increase in rates of BP diagnoses occurred in parallel with a rise of prescription rates of antipsychotic medication [4]. Diagnostic practice seemed to be the most likely explanation for the observed increase in BP diagnoses [5] and irritability seems to have been at the heart of this problem. Based on the premise that mania may present differently in youths than in adults, some researchers suggested that chronic, non-episodic irritability is a core characteristic of BP in young people (e.g. [6].). This is inconsistent with the DSM-IV criteria for BP, which specify the need for a “distinct period of abnormally and persistently elevated, expansive or irritable mood”. Moreover, irritable mood is relatively less specific to BP in the DSM-IV. It requires the presence of four additional symptoms for the diagnosis to be made, compared with three or more for elevated or expansive mood.

### Longitudinal outcomes and family history of irritability

Irritability, although not formally defined in the DSM-IV, is a mood symptom present in the criteria for a large number of psychiatric disorders and refers to easy annoyance and touchiness that can manifest in anger and temper outbursts [1]. Depending on the stringency of threshold, prevalence rates of irritability range from 3 % for severe, chronic irritability strictly defined in the Great Smoky Mountain study [7] to 20 % in the Isle of Wight study [8]. In response to the bipolar controversy, Leibenluft et al. [9] conceptualised chronic irritability as severe mood dysregulation (SMD), a category generated to allow for testable comparisons with BP. SMD is characterised by chronic and severe irritability with frequent and developmentally inappropriate temper outbursts, along with negatively valenced mood in between outbursts.

Follow-up studies contradicted the notion that severe irritability is an early manifestation of mania. Children with SMD at the age of 10 years suffered from unipolar depression, but not BP at the age of 18 years [7]. Stringaris et al. [10] reproduced this finding using a 20-year follow-up study. However, these studies were of community samples where the rates of BP are low. Therefore, not finding an association between BP and chronic irritability may have been due to the lack of statistical power. To address this, Stringaris et al. [11] used a referred sample to compare the course of children with SMD and those with BP over a median period of 29 months. The authors found that only one out of 84 (1.2 %) patients with SMD experienced a manic episode over this time period. By contrast, 58 out of 93 (62.4 %) patients with BP had a manic episode over the same time period. Again, such findings argue against the notion that chronic irritability is a characteristic of BP. It will be informative to follow children with SMD further as they pass through the period of maximum risk for BP.

Consistent with these findings, SMD and BP were found to differ in family history [12], with parents of youth with narrow phenotype BP being significantly more likely to be diagnosed with BP (14/42, 33.3 %) than parents of youth with SMD (1/37, 2.7 %).

### Pathophysiological findings

Recently, research has compared whether severe irritability, in the form of SMD, and BP overlap in their pathophysiological mechanisms. SMD and BP youth were both found to display deficits in face emotion labelling. Both patient groups were significantly less accurate in labelling facial emotions [13] and less sensitive in recognising facial expressions of emotions [14], compared to healthy controls. However, SMD and BP differ in the brain mechanisms underlying these emotion labelling deficits—a recent functional magnetic resonance imaging (fMRI) study showed that patients with SMD had lower amygdala activity compared to those with BP during a face emotion processing task [15]. The pattern of amygdala activation in SMD resembled that of young people with depression [16, 17].

Differences between SMD and BP youth have also been identified in studies using the affective Posner task [18], an attention task designed to elicit frustration. Rich et al. [19] showed that although both SMD and BP youth displayed significantly more negative affect than healthy controls in response to negative feedback, patients with SMD differed from those with BP in their event-related potentials (ERPs) [20] and brain activation patterns [19]. The pattern of ERPs following frustration suggested executive attention deficits in the BP, but not SMD group, who were impaired in the initial, “bottom-up” attention regardless of emotional context. These findings are consistent with neuroimaging data: in response to blocked goal attainment on the frustration task, relative to SMD youth, BP youth displayed greater activation in the superior frontal gyrus (SFG) [19], a region implicated in the regulation of executive attention [21]. Coupled with neuroimaging techniques, frustration tasks offer a promising way of disentangling SMD from BP, by suggesting that the seemingly similar behavioural manifestations of irritability may have different neural substrates.

Related findings come from a series of studies using response reversal paradigms, where participants have to modify their responses to match the environmental changes. These are usually changes as to which stimulus is rewarded and which one is not. Poor performance on these tasks was suggested to lead to frustration [22]. Although response reversal deficits were more often found in young people with BP (e.g. [23]), a recent study found that both SMD and BP youth showed deficits on response reversal with compound stimuli [24]. Emerging data suggest that SMD and BP youth may differ in the underlying pathophysiology of response reversal deficits, with SMD but not BP youth showing reduced inferior frontal gyrus (IFG) activation in response to errors on the task relative to healthy controls [25]. Of note, the same study found both SMD and BP youth to have reduced task-related caudate activation compared to healthy controls.

Taken together, these findings suggest that pathophysiological mechanisms differ between SMD and BP youth; however, they also show that there are shared pathways between the two conditions. The next important step will be to compare severe irritability with other childhood psychopathology, such as attention deficit hyperactivity disorder (ADHD), and establish their distinguishing neurobiological features. It will also be important to take a dimensional view of irritability. New measures, such as the Affective Reactivity Index (ARI) [26], could be useful in studying the brain correlates of irritability intensity. This will further help to understand the mechanisms of irritability and may be useful for diagnostic purposes.

An important clinical problem is irritability in young people with autism spectrum disorders (ASD). For good reasons, a diagnosis of ASD was an excluding factor in studies on mood dysregulation mentioned above, and therefore irritability in ASD has been understudied. Emerging evidence points on important differences between how mood problems present in ASD compared to typically developing youth. For example, in contrast to studies of typically developing young people with SMD [13, 24], similarly defined severe mood problems in ASD youth were not associated with deficits in executive function or emotion recognition (except for identifying the facial expression of surprise) [27]. Future research should determine which aspects of ASD affect the presentation of mood problems in this clinical group. This may inform the diagnosis and treatment of irritability not only in the ASD population, but potentially also in typically developing youth who display some autistic-like traits, such as rigidity or social interaction difficulties.

### Implications for assessment and clinical guidelines

The assessment of irritability has until recently lagged behind other research in the field. In most studies, irritability was measured using items from the oppositional defiant disorder (ODD) section of interviews [28]. More recently, a concise instrument to measure irritability, the ARI has been developed and validated in clinical samples [26]. SMD can be assessed using a specially developed module [11] of the Kiddie Schedule for Affective Disorders and Schizophrenia for School Age Children (K-SADS) [29].

### Treatment implications

In contrast to classical BP [30], chronic non-episodic irritability lacks appropriate evidence-based treatments, largely due to its unclear diagnostic and aetiological status. Extrapolating treatment options from classical BP to SMD, relying on the premise that severe, chronic irritability constitutes a developmental manifestation of BP, may be harmful. For example, it is unclear whether atypical antipsychotics, such as risperidone, used in the treatment of irritability in autism [31], would be a viable treatment option for irritability in typically developing youth with SMD. Lithium was already found to be ineffective in SMD [32]. A trial of a serotonin reuptake inhibitor in children with SMD is ongoing (Leibenluft, personal communication). Clinicians will of course want to treat other comorbid disorders, particularly ADHD, in children presenting with severe irritability. Treatment with methylphenidate was found to reduce symptoms of oppositionality in children [33], and in our practice and according to anecdotal evidence, it also seems to reduce irritability in those with ADHD. In our experience, cognitive-behavioural therapy (CBT) appears to be a well-tolerated and often effective treatment for children with irritability [34]; however, no trial data are yet available about the effects of psychological therapy on children’s irritability.

## Short-lived episodes of mania-like symptoms

### Overview

The second important issue when comparing adult and paediatric BP concerns the duration criteria for mania in DSM-IV in youth. Adults meet DSM-IV criteria for mania if symptoms have been present for at least 7 days and for hypomania if symptoms have been present for 4 days. In adults, shorter episodes (1–3 days) of hypomanic symptoms are thought to be manifestations of bipolar spectrum; individuals with such shorter episodes do not seem to differ from those with episodes of typical duration (>4 days) on a range of clinical validators [35]. A question has been raised whether duration criteria of 4–7 days are too stringent for youth and whether a BP diagnosis should be made in those with more brief duration hypomania.

### Brief duration hypomania and bipolar disorder

Young people who meet the DSM-IV symptom and impairment criteria, but present with symptoms of mania for less than 4 days, frequently receive the diagnosis of bipolar disorder-not otherwise specified (BP-NOS). Cases of children with BP-NOS are indistinguishable from those with classical BP in age of onset, duration, severity, comorbidity, family history and the presence of suicidal ideation and major depression, according to the findings of the course and outcome in bipolar youth (COBY) study [36]. Approximately 25 % of BP-NOS youth developed BP-I or BP-II at a 2-year follow-up period [37] and 38 % reached this diagnosis over 4-year follow-up [38]. However, it is unclear which cases of BP-NOS were most similar to classical BP, because the authors did not distinguish between cases with very short (less than 1 day) episodes and cases with episodes closer to the DSM-IV duration limit of 4 days.

In a community sample, BP-NOS remained associated with functional impairment after adjusting for the effect of other psychopathology [39]. These findings and the rarity of classical BP in youth (0.1 % compared to 1.1 % in a community sample [39]) suggest that short-lived episodes of mania may be a developmental presentation of BP. However, this hypothesis is inconsistent with the finding that prevalence and duration of BP-NOS were not moderated by participants’ age [39]. Furthermore, lack of agreement between child- and parent-reported episodes in this sample raises doubt over the validity of combining information about youth BP symptomatology across informants.

### Implications for assessment and clinical guidelines

There are no official guidelines about short-lived episodes of mania-like symptoms in youth. Clinicians can ascertain shorter duration episodes of mania as part of their usual interview for BP. A module of the K-SADS has been developed and used in the COBY studies that ascertains short episodes [40]. A section of the Development and Wellbeing Assessment (DAWBA) [41] has also been used to study short-episode durations in epidemiological studies.

### Treatment implications

No evidence-based treatments have yet been developed for brief episodes of mania-like symptoms in youth. In addition, because the relationship of such episodes to classical mania remains unclear, extrapolating evidence from what is known about BP may be problematic. It is possible that very short episodes of mania-like symptoms may in fact reflect exacerbations of underlying chronic externalising disorders, such as ADHD or ODD [42], which would call for a different treatment. The two presentations may be potentially difficult to differentiate for young people and their parents who undergo diagnostic interviews. Clinicians will be more likely to treat children with mania-like symptoms using antipsychotic or mood stabilising medication if their episodes last for at least 2–3 days and lead to impairment. By contrast, the use of antipsychotic medication in children with very short-lived (less than 1 day) episodes should be treated with great caution.

## Implications for nosology and outstanding questions

### Chronic irritability

The distinction between severe, non-episodic irritability and classical BP has important implications for diagnosis. In response to concerns about a possible over-diagnosis of BP in youth, the DSM-5 Task Force proposed a new diagnostic category of disruptive mood dysregulation disorder (DMDD) [43]. The criteria for DMDD resemble those for SMD [9], although DMDD does not include symptoms of hyperarousal.

The DSM-5 proposal met with some criticism concerning DMDD’s specificity. Axelson et al. [44] pointed out that irritability is a symptom present in a number of disorders within the DSM-IV, such as major depressive disorder (MDD), generalised anxiety disorder (GAD), ODD and ADHD which would imply that DMDD irritability may not be specific. However, this is not a sufficient argument against the category of DMDD. Inattention and concentration difficulties also occur as part of the criteria of different psychiatric disorders, but inattention is still usefully distinguished within ADHD. Moreover, the occurrence of irritability as part of the criteria of several different psychiatric disorders may be a mistake of previous classifications and should not be treated as a given. It is an empirical question whether several of the current diagnoses that feature irritability in their criteria could stand without it.

It has also been suggested to use irritability as a course specifier for other psychiatric disorders, for example ODD, instead of introducing DMDD. Even in such a case, clinicians would still have to make a categorical judgement about the presence of clinically relevant irritability, a process not much different from deciding whether DMDD is present or not. Moreover, introducing a specifier would not provide a diagnostic home for those children who suffer from irritability, but have no other diagnosable psychiatric disorder. An alternative solution would be to treat irritability as a dimension, rather than a category. Clinicians would determine where their patients lay on a spectrum of irritability severity in the same way that internists determine the levels of hypertension in their patients. This is an attractive model and new measures exist [26] that could be useful in this process. However, there are obvious difficulties. Clinicians will require thresholds to help them make binary (“treat or not treat”) decisions and clearly more research is required to establish clinically-relevant irritability cut-offs. Also, introducing dimensions that cut across psychopathology would need to be carefully considered in the context of all other possible symptom dimensions in psychiatry, not just irritability.

Whether to include DMDD as a separate category in the DSM-5 is a complex issue, inevitably linked to more general questions about how best to classify disease [45]. Including DMDD could lead to errors of commission (such as those discussed above); however, not addressing irritability in the new classification would be to continue with an error of omission. The bipolar controversy has taught us that ignoring children’s irritable mood comes at a cost to public health. Any classificatory scheme should at least allow clinicians to record irritability and make it possible to investigate this common mood problem in developmental psychopathology.

### Short-lived episodes of mania-like symptoms

There is a proposal in the DSM-5 to capture episodes with mania-like symptoms of 2–3 days duration under BP-Not Elsewhere Classified (BP-NEC) [46]. Empirical evidence suggests that, at least in youth, such change could have profound implications for the prevalence of mania [39]. Some evidence suggests that at least some children with short-lived episodes of mania-like symptoms may be on a continuum of bipolarity [38], which seems to be consistent with findings in adults [35]. However, the main challenge will be to establish the characteristics of those who are most likely to fall within this bipolarity spectrum and who might benefit from early treatment. What mechanisms determine whether some patients predominantly present with short-lived episodes of mania-like symptoms, while others display full-blown manic episodes? What mediates the transition from short-lived to longer-lasting episodes? Are personal characteristics such as genetic load and comorbid psychopathology important, and what is the contribution of environmental (e.g. occupational or other life-style) factors?

## Conclusions

Despite considerable progress in understanding irritability and short-lived episodes of mania-like symptoms in children, a lot more needs to be done. It would be wrong for nosology to rush ahead of the evidence; equally, the DSM-5 cannot remain blind to the deficits of the current classification. The DSM-5 should provide the conditions for both irritability and short-lived episodes of mania-like symptoms to enter clinicians’ awareness and to become a topic of ongoing clinical research.
